# Microsomal Prostaglandin E Synthase-1 Plays a Critical Role in Long-term Motility Dysfunction after Bowel Obstruction

**DOI:** 10.1038/s41598-018-27230-6

**Published:** 2018-06-11

**Authors:** You-Min Lin, Yu Fu, Shrilakshmi Hegde, Yanbo Tang, Ravi Radhakrishnan, Xuan-Zheng Shi

**Affiliations:** 1 Department of Internal Medicine, University of Texas Medical Branch, Galveston, TX USA; 2 Department of Gastroenterology, The first Affiliated Hospital, Guangxi University of Science and Technology, Liuzhou, Guangxi China; 3 Department of Surgery, University of Texas Medical Branch, Galveston, TX USA

## Abstract

Motility dysfunction is present not only during bowel obstruction (BO), but after obstruction is resolved. Previous studies found that lumen distension associated mechano-transcription of COX-2 and production of PGE_2_ in gut smooth muscle cells (SMC) account for motility dysfunction during obstruction. We hypothesized that PGE_2_ may exert autocrine effect in SMC to induce microsomal prostaglandin E synthase-1 (mPGES-1), which contributes to motility dysfunction after obstruction is resolved. Partial colon obstruction was induced in rats with an obstruction band, which was released 7 days later. Rats were further studied in the post-BO state. Circular muscle contractility of the mid colon (previously distended during obstruction) remained suppressed, and colon transit was impaired in the post-BO state. The COX-2, mPGES-1, and PGE_2_ levels were all increased in the distended bowel during obstruction. However, after obstruction was resolved, COX-2 expression returned to normal, whereas mPGES-1 and PGE_2_ levels remained increased. Expression of mPGES-1 in colon SMC was inducible by stretch or PGE_2_. Administration of mPGES-1 inhibitor Cay 10526 either before or after the release of obstruction normalized PGE_2_ levels and improved motility in the post-BO rats. In conclusion, mPGES-1 plays a critical role in the continuous suppression of motor function in the post-BO state.

## Introduction

Bowel obstruction (BO), mechanical or functional, is a serious health challenge^1,2^. BO accounts for more than 300,000 hospital admissions per year in the United States alone; the aggregate cost for hospital stay is more than $3 billion annually, topping all other gastrointestinal conditions^1,3^. Mechanical obstruction is caused by a physical blockage of the intraluminal passage, which may originate extrinsic to the gut, e.g. adhesions, hernias, or intrinsic to the gut, e.g. carcinoma and diverticulitis^1,2^. Functional obstruction results from neuromuscular dysfunctions such as those in pseudo-obstruction and Hirschsprung’s disease (HD)^4–6^. In HD, the terminal bowel is constricted due to lack of myenteric ganglia, and the segment oral to the constricted segment becomes distended^6,7^.

The current surgical approach for obstruction is to release blockage as in mechanical BO, or remove constriction as in HD, but to leave alone the distended oral segment in the gut^6,8^. However, many patients suffer motility dysfunction not only during the course of obstruction, but after the obstruction is surgically resolved^9–15^. Among children with a history of HD, 50~70% suffer long-term gastrointestinal (GI) symptoms such as constipation, incontinence, abdominal distention, and enterocolitis in the first 5 to 10 years after pull-through surgery^12,13^. The latest data showed that gut dysfunction continues into adulthood after surgical release of obstruction in childhood, as 30% of HD patients (9% in control) were reported to suffer from constipation and motility dysfunction when they became adults at a mean age of 43^14^. In the upper gut, pyloromyotomy to correct infantile pyloric stenosis also has long-term effects on gastric sensory and motor functions^16,17^. The tonic and phasic contractions in the stomach are impaired for more than 10 years after surgical resolution of the outlet obstruction^17^. Motility dysfunction is responsible for symptoms such as bloating, nausea, vomiting, and constipation^2,8,12–14,18^. However, what accounts for the long-term motility dysfunction following release of obstruction (post-BO) is not known. It is important to elucidate the underlying mechanisms of long-term motility dysfunction after obstruction, so that therapeutic targets may be identified.

Our recent studies in a rodent model of partial colon obstruction found that lumen distention during BO leads to mechanical stress-induced gene expression (mechano-transcription) of cyclo-oxygenase-2 (COX-2) in the distended oral segment, but not in the non-distended aboral segment^18^. We found that mechano-transcription of COX-2 and COX-2 -derived prostaglandins (PG) in the distended bowel plays a critical role in the suppression of motility function during the course of BO^18,19^. Lin *et al*. found that COX-2 inhibitor may have the therapeutic potential in the management of motility dysfunction and associated symptoms during BO^20^. However, it remains to be determined whether distention-induced COX-2 and other mechanically sensitive molecules remain up-regulated after obstruction is resolved, and whether expression of these mechanically sensitive genes exert autocrine or paracrine actions to further alter gene expression and gut functions in the previously distended bowel segment.

Among the COX-2 –derived prostaglandins, PGE_2_ is the most abundant in the gut, and has profound impacts on motility and other gut functions in obstructive conditions^20–22^. PGE_2_ acts on gut SMC through E-prostaglandin (EP) receptors, i.e. EP1 – EP4^22^. We found that PGE_2_ impairs colonic circular smooth muscle contractility in BO mainly through EP2 and EP4 receptors^20^. PGE_2_ is generated from PGH_2_, a direct COX product, by PGE synthase (PGES). There are three isomerases of PGES. Microsomal PGES-1 (mPGES-1) is inducible, whereas mPGES-2 and cytosolic PGES-1 (cPGES-1) are constitutively expressed^23–25^. As COX-2 inhibitors have toxic side effects and show non-discriminating inhibition on synthesis of all PGs, mPGES-1 has been considered a promising therapeutic target for PGE_2_ related pathologies^22–25^. However, the regulatory mechanisms and pathophysiological role of mPGES-1 in the gut are not well understood.

In the present study, we first established a rodent model of post-BO, in which partial colon obstruction was induced with an obstruction band^18^, and resolved by releasing the band 7 days later. We found that colon smooth muscle contractility remained significantly suppressed after obstruction is released (post-BO state). Colon transit rate was slower in the post-BO rats than in the sham control. The main objective of the study was to investigate the mechanisms underlying motility dysfunction in the post-BO state and to identify potential therapeutic targets to improve motility function after obstruction is resolved. We found that although mechanical stress-induced expression of COX-2 returned to normal level after obstruction is resolved, mPGES-1 expression and PGE_2_ production remained elevated in the post-BO state. We tested the hypotheses that COX-2 –derived PGE_2_ during obstruction further up-regulates expression of mPGES-1, and that mPGES-1 may play a critical role in the continued production of PGE_2_ and prolonged motility dysfunction after obstruction is resolved.

## Methods

### Rodent model of post-BO and experimental design

The Institutional Animal Care and Use Committee at University of Texas Medical Branch approved all the procedures performed on the animals. Experiments were performed in accordance to the Guide for the Care and Use of Laboratory Animals of the National Institutes of Health, USA.

Male Sprague-Dawley rats weighing 225–275 g (Harlan Sprague Dawley, Indianapolis, IN) were used in the study. The rats were housed in a controlled environment (22 °C, 12-h:12-h light/dark cycle) and allowed food and water ad libitum. Partial colon obstruction was prepared as previously described^18,20,26^. To make post-BO model, colon obstruction was maintained with an obstruction band placed in the distal colon (3–4 cm oral to the end) until the band was released 7 days later. In brief, 7 days after initial operation, sham and obstruction rats were anesthetized with 2% isoflurane inhalation by an E-Z Anesthesia vaporizer (Palmer, PA). A midline laparotomy was made, and the obstruction band was released in the obstruction rats. The rats in post-BO state were kept for up to 6 weeks until euthanasia. The sham control rats underwent the similar surgical schedule with two laparotomy operations, except that obstruction band was installed but released immediately in the first operation.

In some experiments, in order to determine the possible roles of mPGES-1 and COX-2 in post-BO motility *in vivo*, animal groups were randomly assigned to include sham and Post-BO rats each with vehicle (DMSO) or mPGES-1 inhibitor CAY10526 (Cayman Chemical, Ann Arbor, MI) at 2.5 mg/kg i.p. daily^25,27^, or COX-2 inhibitor NS-398 at 5 mg/kg i.p. daily^18,20^, starting on the day when obstruction band was released. Rats were studied 7 days after obstruction was released (post-BO day 7).

In another experiment, mPGES-1 inhibitor CAY10526 was administered during the 7-day period of obstruction at 2.5 mg/kg i.p. daily. Obstruction was released and rats were studied 7 days on post-BO day 7.

### Tissue collection

The mid colon segment (~3 cm in length) oral to the original site of obstruction was collected in fresh carbogenated Krebs buffer (in mmol/l: 118 NaCl, 4.7 KCl, 2.5 CaCl2, 1 NaH2PO4, 1.2 Mgcl2, 11 d-glucose, and 25 NaHCO3). The mucosa/submucosa and muscularis externa layers were separated by microdissection as described previously^18–20^. The tissue samples were processed immediately for muscle bath experiments or stored at −80 °C for molecular studies. Full thickness samples were saved in 10% buffered formalin for immunohistochemistry study.

### Immunohistochemistry study

Immunohistochemistry staining of COX-2 was performed as described previously^18,26^ on formalin-fixed, paraffin-embedded colon segments in sham control and in rats with obstruction and in post-BO state. Sections at 4-μm thickness were blocked with 5% normal goat serum in PBS for 20 min at room temperature, and incubated with the rabbit anti-COX-2 antibody (1:200, Cayman Chemical) and a biotin-conjugated anti-rabbit secondary antibody (Vector Laboratories, Burlingame, CA). After incubation with avidin-biotin complex (Vector kit, Vector Laboratories), the sections were stained in diaminobenzidine tetrahydrochloride with 0.03% hydrogen peroxide. As a negative control, sections of the same specimens were processed by the same method but omitting the anti-COX-2 primary antibody.

### RNA preparation and quantitative RT-PCR

Total RNA was extracted from tissues or cells using the Qiagen RNeasy kit (Qiagen, Valencia, CA), and reverse transcribed with SuperScript III First-Strand Synthesis System (Invitrogen, Carlsbad, CA)^18–20,26^. Real-time quantitative PCR was performed using the Applied Biosystems 7000 real-time PCR system (Foster City, CA) as described^18–20,26^. The TaqMan primers and probes for detection of rat COX-2, cPGES-1, mPGES-1, and mPGES-2 mRNAs were purchased from Invitrogen. The fold change relative to control was calculated with the comparative Ct (ΔΔCT) method with endogenous reference 18S rRNA as the normalizer.

### Enzyme immunoassay of PGE_2_

Rat colonic muscularis externa tissue was homogenized in cold PBS buffer (in mmol/l 137 NaCl, 2.7 KCl, 10 Na2HPO4, KH2PO4, pH 7.4) supplemented with protease inhibitors. PGE_2_ in the extraction was measured with the PGE_2_ enzyme immunoassay kit purchased from Cayman Chemical by following the manufacturer’s protocols^18,20,21^.

### Muscle bath experiments

Colon segments 1∼2 cm oral to the original site of obstruction were opened along the mesenteric border, and pinned flat in a Petri dish with Sylgard base in carbogenated Krebs buffer. The mucosa/submucosa layers were separated and discarded by microdissection. The smooth muscle strips (3 mm × 10 mm) were mounted along the circular muscle orientation in individual muscle baths (Radnoti Glass, Monrovia, CA) filled with 10 ml of carbogenated Krebs solution at 37 °C. The contractile activity was recorded as previously described^18,19,28–30^ with Grass isometric force transducers and amplifiers connected to Biopac data-acquisition system (Biopac Systems, Goleta, CA). The muscle strips were equilibrated in the muscle bath under 1 g tension for 60 min at 37 °C before they were tested for contractility. Muscle contractility was tested in response to acetylcholine (ACh) (10^−6^ to 10^−2^ M) and to KCl (62.5 mM). The strips were left to equilibrate for 15 to 20 min between additions of different concentrations of ACh or KCl. When the contractile response to KCl was tested, the concentration of KCl (62.5 mM) in the Kreb’s buffer was increased by the equimolar replacement of NaCl. The contractile response was quantified as the increase in area under contractions (AUC) during 4 min after the addition of ACh or KCl over the baseline AUC during 4 min before the addition of ACh or KCl.

### Measurements of colon transit

The colonic transit rate in sham and post-BO rats was determined by the geometric center method as described previously^20,28^. In brief, a bolus of 1.5 ml of 1.5% methylcellulose (Fisher Scientific, Fair Lawn, NJ) containing 0.75 mg non-absorbable phenol red was injected into the colon via a catheter implanted earlier. Rats were euthanized by CO_2_ inhalation 60 min later. The whole colon was removed immediately and divided into six segments of equal length. Each segment, along with its intraluminal contents, was placed in 25 ml of 0.1 N NaOH and homogenized. The homogenate was kept at room temperature for 1 h, and 1 ml of the supernatant was added to 0.1 ml of 20% trichloroacetic acid solution to precipitate proteins. After centrifugation at 10,000 g for 15 min, 1 ml of 0.5 N NaOH was added to the supernatant. The amount of phenol red was determined by measuring the absorption at 550 nm with a Biophotometer Plus (Eppendorf North America, Westbury, NY). Colon transit was calculated as the geometric center of distribution of phenol red as follows: geometric center = ∑ (counts of phenol red per segment × segment number)/∑ (counts of phenol red per segment).

### Primary culture of rat colon SMC and *in vitro* mechanical stretch

Rat colonic circular SMC (RCCSMC) were isolated as described previously^18,26,31,32^. In brief, the circular muscle tissue in 0.5 × 0.5 cm2 size was incubated in sterile HEPES buffer (in mmol/L: 120 NaCl, 2.6KH2SO4, 4KCl, 2CaCl, 0.6MgCl2, 25 HEPES, 14 glucose, and 2.1%essential amino acid mixture, PH 7.4) with 1.5 mg/ml collagenase (type II, 319 U/mg; Worthington, Freehold, NJ) and 1.0 mg/ml soybean trypsin inhibitor (Sigma-Aldrich) for 45 min at 31 °C. At the end of digestion, tissue pieces were incubated in fresh buffer without digestion enzymes. The spontaneously dispersed cells were collected and cultured in DMEM supplemented with 10% fetal bovine serum (FBS) in the presence of 100 U/ml of penicillin G, 100 µg/ml streptomycin sulfate, and 0.25 µg/ml amphotericin B (invitrogen). The culture medium was changed every 3 days^32^.

Primary culture was allowed to grow for 8–10 days until cells were confluent. The cells were then seeded at 8 × 10^4^ cells/well in six-well BioFlex culture plates coated with type I collagen (Flexcell International, Hillsborough, NC). Cells were allowed to grow to ∼80% confluence before being subjected to DMEM/1% FBS for overnight prior to mechanical stretch^18,26^ via a FX-4000 Flexercell Tension Plus System (Flexcell International). This computer-regulated bioreactor applies multi-axial strain to cultured cells. Through vacuum pressure, cultured cells were stretched on the flexible membrane plates. Cells incubated in parallel under identical conditions but without exposure to stretch served as controls.

### Statistical analysis

Data points are expressed as means ± SEM. Statistical analysis was performed by analysis of variance with non-repeated measures (by Student-Newman-Keuls test) for comparisons of multiple groups and Student’s t-test for comparisons of two groups. A *p* value of ≤0.05 was considered statistically significant.

### Data availability

The datasets generated during and/or analyzed during the current study are available from the corresponding author on reasonable request.

## Results

### Motility dysfunction in post-BO state

**Figure 1:**
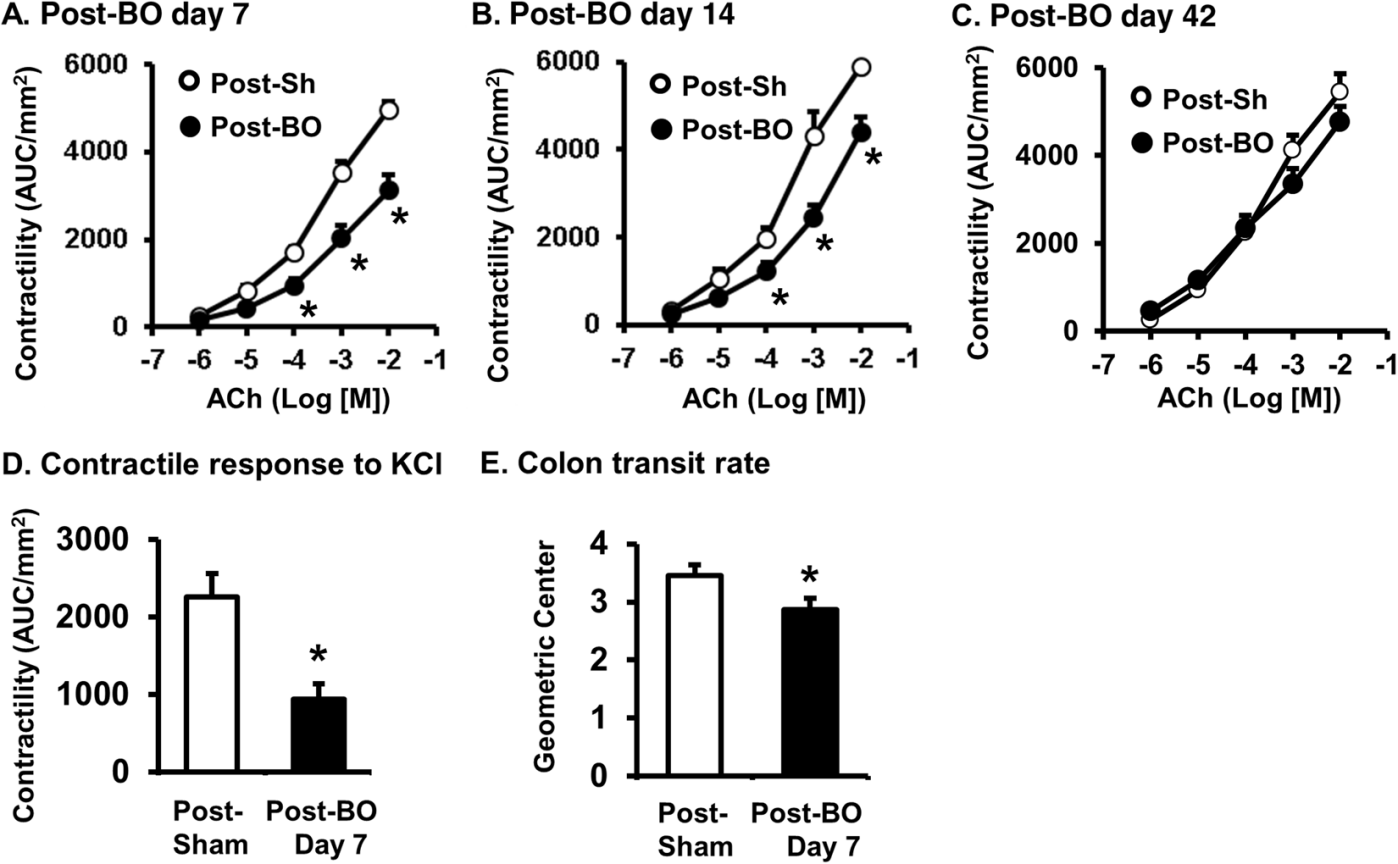
Circular smooth muscle contractility (**A**–**D**) and colon transit (**E**) in the post-BO state. To determine muscle contractility, colonic circular muscle strips were prepared from the mid colon of post-sham and post-BO rats. The mid colon was taken, as it was where mechanical distention occurred during obstruction. Note that colonic circular muscle contractility to ACh was suppressed on day 7 (n = 6, **A**) and day 14 (n = 5, **B**), but recovered on day 42 (n = 5, **C**) in post-BO state. Muscle contractile response to KCl was also reduced in post-BO state (post-BO day 7, n = 5) (**D**). Colon transit was measured in a separate study (**E**) on different rats from those used for muscle bath. Note that colon transit rate is slower in post-BO rats (post-BO day 7, n = 6), compared to post-sham (7 day after operation, n = 5). *p < 0.05 vs Post-sham.

Previous studies found that colonic motility was suppressed significantly during the course of obstruction in the rat model of BO^18^. To determine if motility function remains suppressed in the post-BO state, we first measured colon circular smooth muscle contractility in response to cholinergic muscarinic receptor agonist ACh in the sham and post-BO rats 7, 14, and 42 days after obstruction was released. Compared to sham, muscle contractility of the mid colon (previously distended during BO) remained significantly suppressed 7 and 14 days after obstruction was released (post-BO day 7 and 14, Fig. 1A,B). However, by post-BO day 42, the smooth muscle contractility recovered to the levels of the sham controls (Fig. 1C).

To determine if muscle contractility is affected in post-BO state in a receptor-independent manner, we also tested the muscle contractile response to KCl (62.5 mM), as KCl bypasses G-protein coupled receptors i.e. muscarinic receptors and induces smooth muscle contraction by depolarizing membrane potential and activating voltage-gated Ca^2+^ channels^28,30^. We found that the contractile response to KCl was also significantly reduced in post-BO rats (Post-BO day 7, Fig. 1D). This data indicates that the suppressed contractile response in post-BO state may involve inhibition of the excitation-contraction coupling mechanisms downstream to receptor activation^30^.

We also measured colon transit rate in sham and post-BO rats (post-BO day 7) (Fig. 1E). It was found that the colon transit rate was significantly delayed in the post-BO rats compared to sham controls, as the geometric center was 2.87 ± 0.19 in the post-BO rats and 3.46 ± 0.18 in the sham control (N = 5 and 6 in sham and post-BO groups, respectively. p < 0.05 vs. sham). These data suggest that colon motility function was impaired significantly after obstruction was resolved.

### COX-2 expression and PGE_2_ production in BO and post-BO

**Figure 2:**
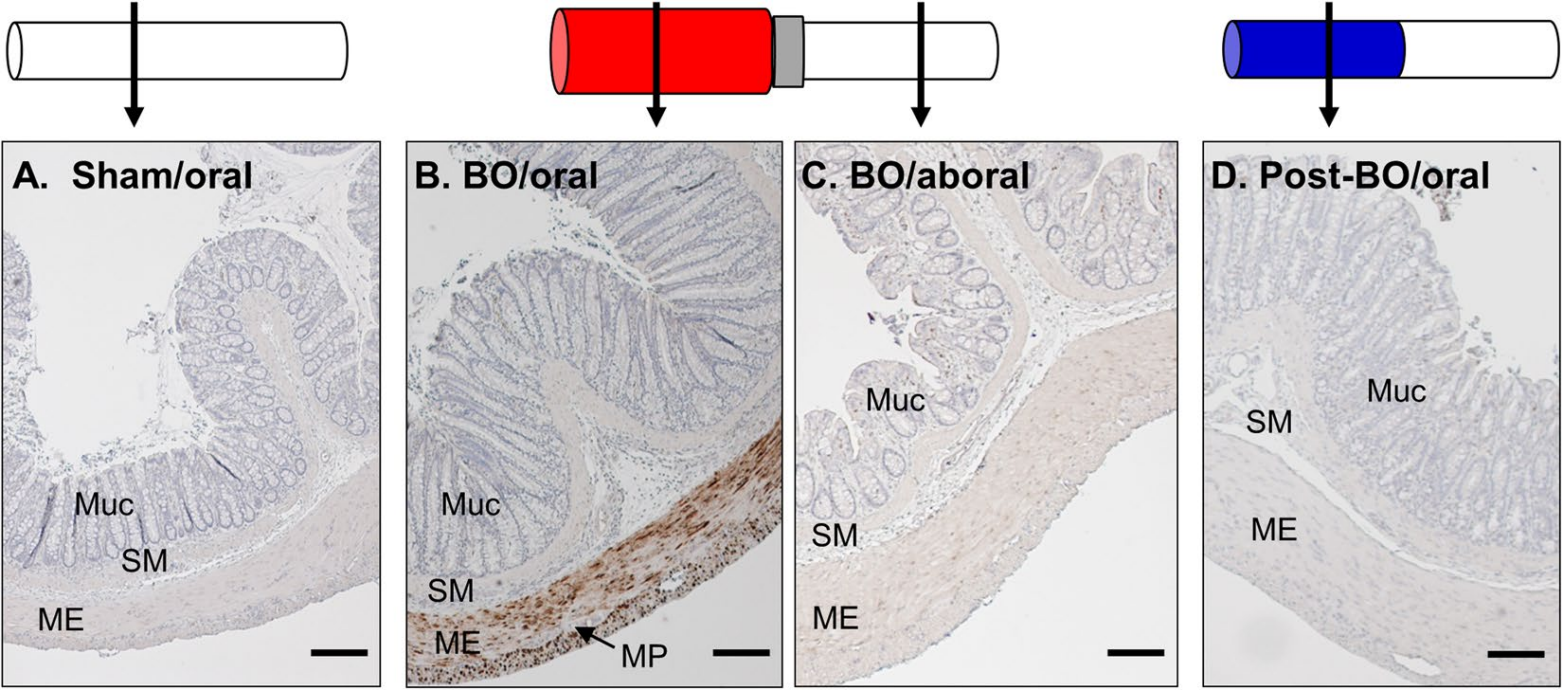
Immunohistochemistry detection of COX-2 expression in BO and post-BO states. Cross-section colon samples were taken from rats in sham (**A**), BO (day 7. (**B**) segment oral to obstruction. (**C**) segment aboral to obstruction), and Post-BO (post-BO day 7, **D**), and stained for COX-2 (in brown). Note that COX-2 expression was detected only in the smooth muscle of the mechanically distended mid colon during BO. Muc, mucosa. SM, submucosa. ME, muscularis externae. MP, myenteric plexus. Images are representatives of 4 or 5 independent experiments in each group. Scale bar = 50 µm.

**Figure 3:**
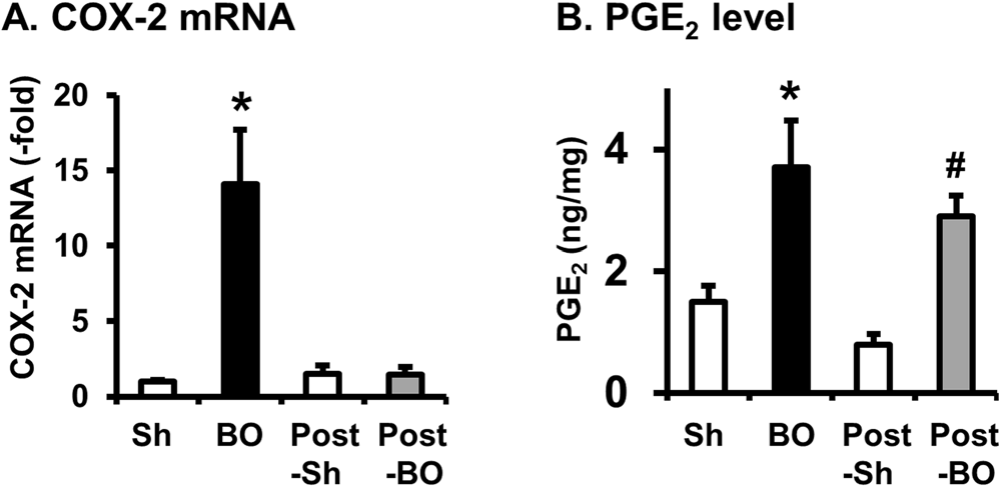
COX-2 mRNA expression (**A**) and PGE_2_ production (**B**) in BO and post-BO states. To detect COX-2 mRNA (**A**) and PGE_2_ production (**B**) colonic muscularis externae was taken from the mid colon of rats in sham (Sh, 7 days after original operation) and BO (day 7 of obstruction), and in Post-sham (7 days after second operation) and Post-BO (day 7 of post-BO state). N = 5 in each group. *p < 0.05 vs. sham; ^#^p < 0.05 vs. Post-Sh.

Our previous study^18^ found that mechanical stress-induced COX-2 expression and COX-2 derived PGE_2_ production play a critical role in the suppression of muscle contractility during obstruction. Consistent with the earlier findings, we noticed that the expression of COX-2 protein and mRNA was significantly induced in colonic smooth muscle of the distended mid colon during obstruction (Figs 2 and 3A). However, COX-2 expression returned to normal levels after obstruction was resolved (Fig. 2). By day 7 of the post-BO period, the COX-2 mRNA level in the colonic muscularis externae was not significantly different from the sham control (Fig. 3A). Immunohistochemistry study revealed that COX-2 was induced in the SMC of the distended colon during obstruction (Fig. 2). However, no COX-2 expression is detectable in the post-BO state in the originally distended colon segment (Fig. 2).

Interestingly, the levels of PGE_2_ in the colonic muscularis externae were increased not only during obstruction, but remained significantly higher in the post-BO rats than in the sham controls (3.6 ± 0.4 vs 1.1 ± 0.2 ng/mg. N = 5 each group. *p* < 0.05) (Fig. 3B).

### PGES expression in BO and post-BO

**Figure 4:**
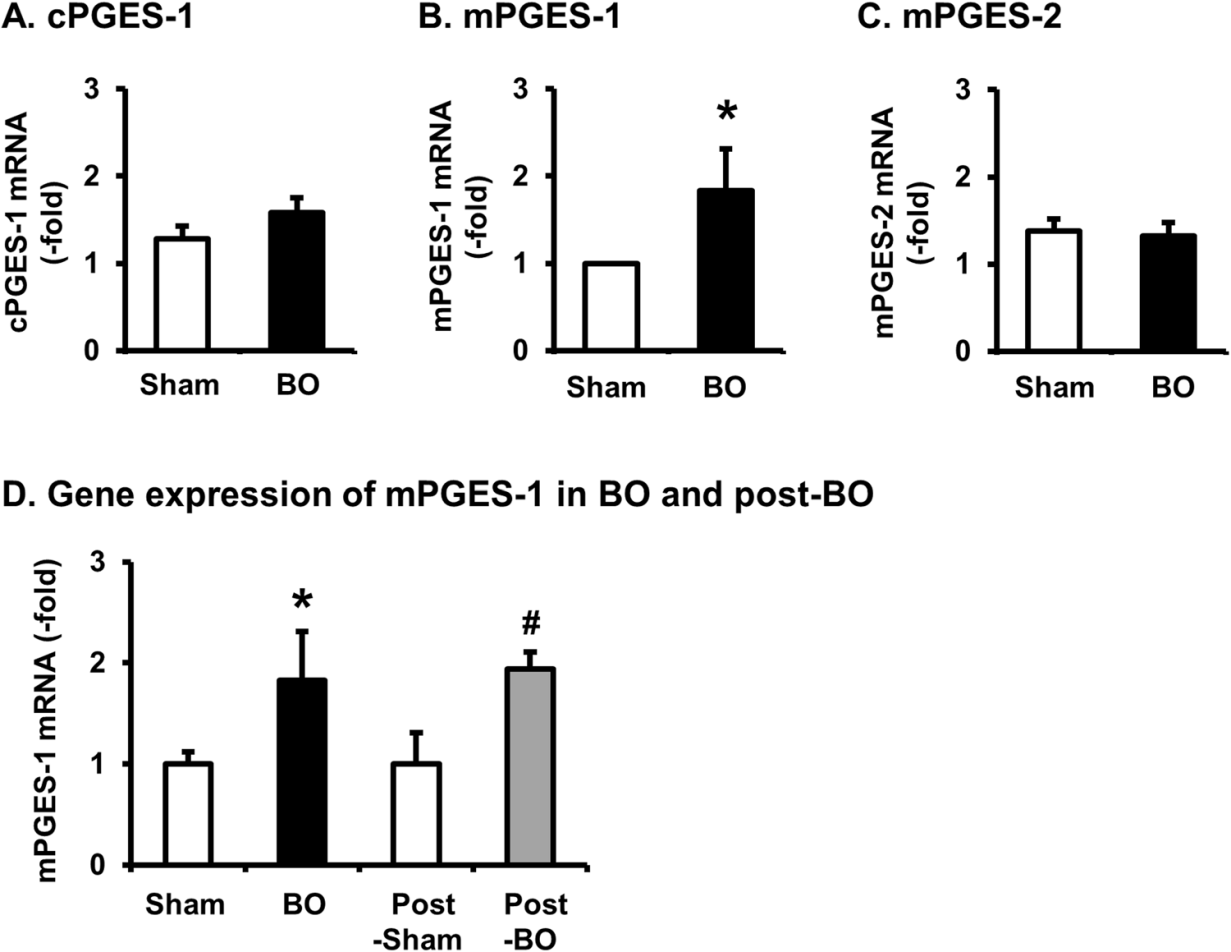
Expression of cPGES-1, mPGES-1, and mPGES-2 mRNAs in colonic muscularis externae in BO (day 7) and post-BO (day 7). (**A**–**C**) The muscularis externae tissues were obtained from the mid colon of sham rats and the distended colon segment (mid colon) oral to obstruction in BO (day 7) rats for detection of mRNA expression of cPGES-1 (**A**), mPGES-1 (**B**), and mPGES-2 (**C**). (**D**) Colonic muscularis externae samples were obtained from the mid colon of rats in sham (Sh) and BO (day 7 of obstruction), and in post-sham (7 days after 2^nd^ operation) and Post-BO (day 7 of post-BO state) for detection of mPGES-1 mRNA. N = 5 each group. *p < 0.05 vs. sham. ^#^*p* < 0.05 vs. Post-Sham.

We sought to determine the mechanisms underlying the persistent increase of PGE_2_ in the post-BO state. As production of PGE_2_ is dependent not only on COX, but on PGES, we determined the mRNA expression of three PGES isomerases in the muscularis externae of the colon. The expressions of mPGES-2 and cPGES-1 were not altered in the distended colon during obstruction (Fig. 4A–C). However, the expression of mPGES-1 mRNA was significantly increased by 1.8 ± 0.4 -fold in the distended segment in BO, compared to sham controls (N = 5 each group. p < 0.01 vs. sham) (Fig. 4B). The expression of mPGES-1 mRNA was not altered in the non-distended aboral segment of obstruction rats (data not shown). More importantly, the mPGES-1 mRNA expression remained up-regulated in the mid colon of post-BO rats (day 7, N = 5), compared to sham (N = 5, p < 0.01 vs. sham) (Fig. 4D).

### Regulation of mPGES-1 gene expression by PGE_2_ and mechanical stretch

The underlying mechanisms for up-regulation of mPGES-1 during obstruction and in the post-BO state were not understood. We tested the hypothesis that both mechanical stress and PGE_2_ may induce the expression of mPGES-1 in colonic SMC. In the *in vivo* model of obstruction, both mechanical stress and increase of PGE_2_ are present during obstruction^23^. We thus chose to test the hypothesis *in vitro* in primary cultures of rat colon SMC, as mechanical stress and PGE_2_ can be applied separately in the *in vitro* settings.

**Figure 5:**
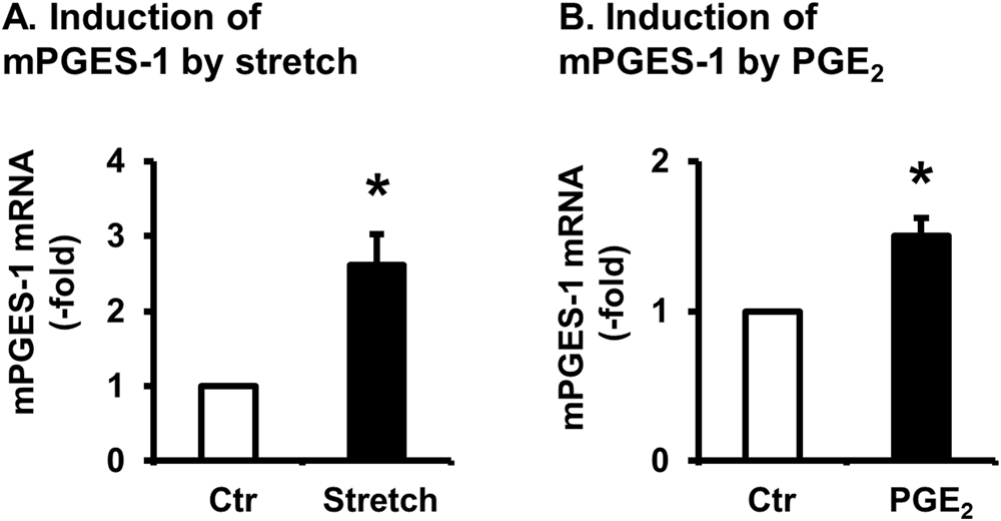
Mechanical stress (**A**) and PGE_2_ (**B**) regulate mPGES-1 gene expression in colon SMC. Primary cultures of rat colonic circular smooth muscle cells (SMCs) were stretched by 18% elongation (**A**), or treated with exogenous PGE_2_ (10^−7^ M, **B**) for 3 hrs. Cells were harvested for detection of mPGES-1 mRNA. N = 4 independent experiments in mechanical stretch (**A**) and PGE_2_ treatment (**B**). *p < 0.05 vs. Ctr.

To test the effect of mechanical stress, primary culture of rat colonic SMC was stretched at 18% elongation for 3 h, cells were harvested for quantitation of mPGES-1 mRNA expression. We found that mechanical stretch significantly induced up-regulation of mPGES-1 mRNA expression by 2.6 ± 0.3-fold, compared to no-stretch control (p < 0.05, 4 independent experiments) (Fig. 5A).

In a separate study, primary culture of rat colonic SMC were treated with vehicle or exogenous PGE_2_ (10^−7^ M) for 3 h. We found that PGE_2_ treatment also significantly up-regulated mPGES-1 mRNA by 1.6 ± 0.1 -fold (p < 0.05 vs. control, 4 independent experiments) (Fig. 5B). These experiments demonstrated that expression of mPGES-1 is subject to regulation by both mechanical stretch and by PGE_2_.

### Role of mPGES-1 in motility dysfunction in BO and post-BO states

**Figure 6:**
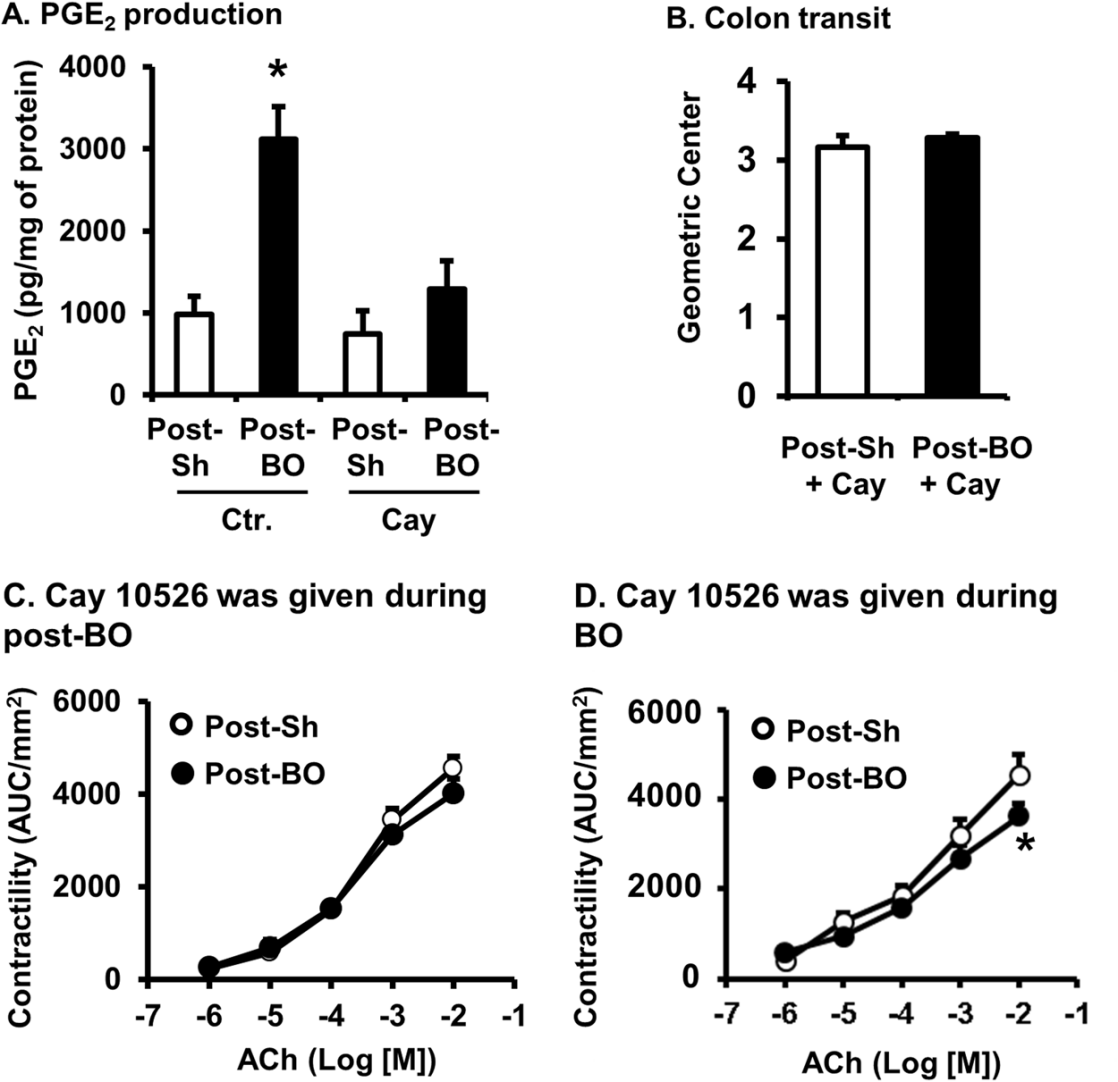
Effects of Cay10526 administrations on motility dysfunction in the post-BO state. (A–**C**) Cay10526 (Cay) was administered daily during the post-BO state. Rats were euthanized on Post-BO day 7 for the determinations of PGE_2_ production in colonic muscularis externae (**A**), colon transit (**B**), and smooth muscle contractility of the mid colon (**C**). N = 5 except for Post-Sh/Cay10526, where N = 4. (**D**) In a separate study, Cay10526 was administered daily during the 7-day course of obstruction (N = 5). Rats were euthanized on post-BO day 7 for measurements of smooth muscle contractility of the mid colon (**D**). *p < 0.05 vs. Post-sham (post-Sh).

Further functional studies were carried out *in vivo* to test the hypothesis that both mechanical stress and PGE_2_ may modulate the expression of mPGES-1 during obstruction, whereas PGE_2_, acting in an autocrine mode, is responsible for up-regulation of mPGES-1 in the post-BO state. The increased mPGES-1 may contribute to the continued increase of PGE_2_ and suppression of motor function after obstruction is resolved. To determine the possible role of mPGES-1 in the production of PGE_2_ and suppression of motility in the post-BO state, we treated rats with daily administration of a specific mPGES-1 inhibitor Cay 10526 at 2.5 mg/kg i.p^24,25,27^. in the post-BO state starting on the day when obstruction was released. We found that Cay 10526 treatment almost completely normalized PGE_2_ levels (Fig. 6A), and significantly improved colon motility in the post-BO rats (Fig. 6B,C). As shown in Fig. 6B, the values of geometric center (GC) were not statistically different between sham (N = 4) and post-BO rats (N = 5) when they were treated with Cay 10526 (Fig. 6B). Cay 10526 treatment in the post-BO state also restored smooth muscle contractility in the previously distended colon (Fig. 6C).

We also determined if administration of Cay 10526 during BO had any effect on motility dysfunction in the post-BO state. For this purpose, Cay 10526 was administered daily during the 7-day course of obstruction only, and colonic circular muscle contractility was measured 7 days after obstruction was released. As shown in Fig. 6D, Cay 10526 treatment during obstruction partially but significantly improved smooth muscle function in the post-BO state (Fig. 6D).

### Role of COX-2 in motility dysfunction in BO and post-BO states

**Figure 7:**
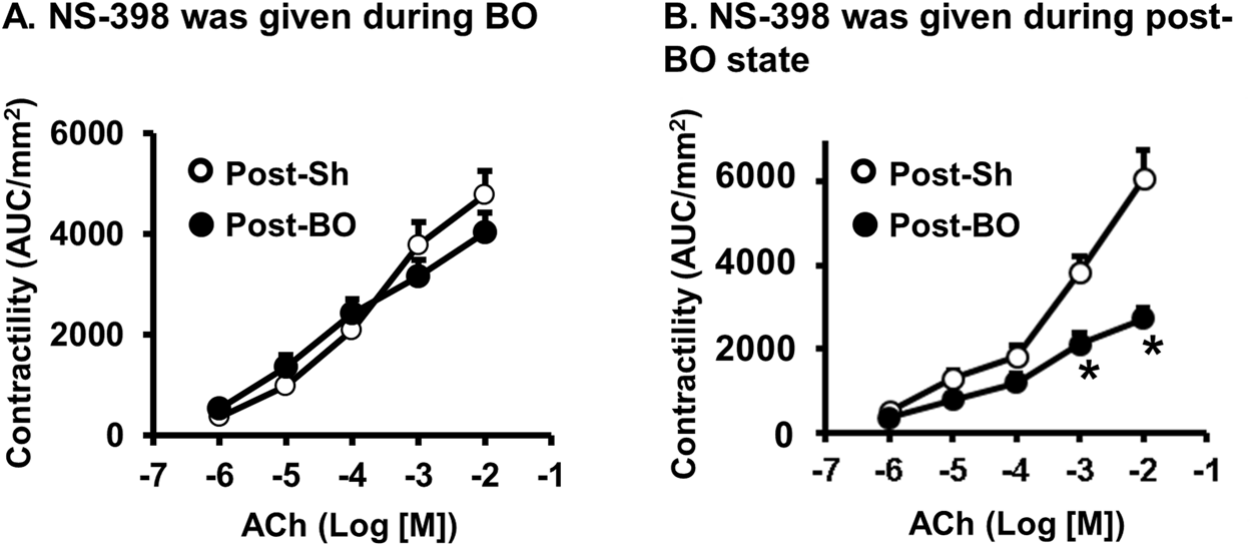
Effects of NS-398 administrations during BO (**A**) and during post-BO state (**B**) on colon smooth muscle contractility in the post-BO state. (**A**) NS-398 was administered daily during the 7-day course of BO. Rats were euthanized on Post-BO day 7 for the measurements of colonic circular muscle contractility of the mid colon. N = 5 in each group. (**B**) NS-398 was administered daily during the post-BO state. Rats were euthanized on Post-BO day 7 for the measurements of muscle contractility of the mid colon. N = 4 in each group. *p < 0.05 vs. sham.

To determine the possible role of COX-2 in motility dysfunction in obstruction and after obstruction is resolved, we administered COX-2 inhibitor NS-398 (5 mg/kg, i.p. daily) during BO and in the post-BO state in separate studies. When NS-398 was administered during BO, it significantly improved muscle contractility in the course of BO^18,21^, and also restored muscle function in the post-BO state (Fig. 7A). However, when NS-398 was administered after obstruction was released (in the post-BO state), it did not significantly improve muscle contractility in the post-BO rats (Fig. 7B).

## Discussion

Motility dysfunctions occur in all stages of bowel obstruction, and may persist after obstruction is resolved^2,9–15^. Immediately after obstruction, gut motor activity is increased in the segment oral to obstruction but suppressed in the segment aboral to obstruction^33^. This transient motility change may result from neuronal mechanisms similar to the physiological peristalsis reflex where food bolus (or luminal accumulation in obstruction) stimulates mechano-receptors in the gut wall to initiate neuronal regulations of ascending excitation and descending inhibition^33–35^. Motor activity in the obstructed bowel soon (in hours) becomes compromised with marked suppression of phasic contractions and muscle tone^18,33,36,37^. Gut motility function continues to be suppressed as long as obstruction is present. The suppressed motility may account for symptoms such as nausea, vomiting, bloating, abdominal distention and constipation^2,12–14,18,30^. We discovered that lumen distention in BO leads to mechano-transcription of COX-2 and other pro-inflammatory mediators in gut smooth muscle^18,31,38,39^, and that the mechanical stress-induced COX-2 plays a critical role in the suppression of muscle contractility and motility function during the course of BO^18,20,38,39^.

**Figure 8:**
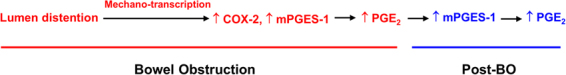
Proposed mechanisms of PGE_2_ production during BO and in the post-BO state. Mechano-transcription of COX-2 and mPGES-1 in SMC accounts for the increased production of PGE_2_ in the distended bowel segment during obstruction. The increased PGE_2_ during BO may further up-regulate the expression of mPGES-1 in the SMC via an autocrine or paracrine mechanism. The PGE_2_-driven up-regulation of mPGES-1 may contribute to the continued production of PGE_2_ and long-term suppression of smooth muscle contractility in the post-BO state.

Although surgical release of obstruction may be the treatment of choice for some patients, especially for those who have complete or complicated obstruction, motility dysfunction does not disappear after obstruction is resolved. Numerous clinical studies documented motility dysfunction in patients with Hirschsprung’s disease or mechanical obstruction after constriction or obstruction is resolved by surgery^9–15^. In the present study, we found that mPGES-1 and m-PGES-1 –derived PGE_2_ may play a critical role in the persistent suppression of gut motility in the post-BO state (Fig. 8). First, PGE_2_ has profound inhibitory effects on gut smooth muscle contractility in the colon^18,20^. We found that PGE_2_ production is increased in the smooth muscle tissue of the obstructed colon and remains increased even after obstruction is resolved. Second, after obstruction was released, mechanical stress was no longer present and the process of mechano-transcription of COX-2 stopped. However, mPGES-1 expression remained up-regulated after obstruction was resolved. More importantly, we found that administration of mPGES-1 inhibitor Cay 10526^25,27^ during the post-BO state blocked the increase of PGE_2_ and restored motility function in the post-BO rats. These studies suggest that mPGES-1 may represent a novel therapeutic target in the management of PGE_2_ related motility dysfunction in obstructive conditions.

Although PGE_2_ is found increased both in obstruction and after obstruction is resolved, the therapeutic strategies to mitigate PGE_2_ related motility dysfunction in BO and post-BO states may be distinctively different. We found that COX-2 inhibitor NS-398, if given during obstruction, is effective in improving motility function during BO and after obstruction is resolved. However, if COX-2 inhibitor is administered in the post-BO state, it does not significantly improve post-BO motility of the colon. To the contrary, treatment with mPGES-1 inhibitor Cay 10526 either during BO or in the post-BO state significantly improved motility function in the post-BO state. Based on the findings in the present and previous studies^23,26^, the differential expression patterns of COX-2 and mPGES-1 in obstruction and in the post-BO state may explain these effects of NS-398 and Cay 10526. Expression of COX-2 was up-regulated during obstruction^18–20^, whereas mPGES-1 expression is increased in both obstruction and in the post-BO state. Our study implies that inhibition of both COX-2 and mPGES-1 during obstruction may be effective as a therapeutic strategy to treat motility dysfunction during BO, and as a prophylactic measure to prevent long-term motility dysfunction occurring after obstruction is resolved. On the other hand, after obstruction is resolved, inhibition of mPGES-1, but not COX-2, may achieve a better therapeutic effect to tackle motility dysfunction (Fig. 8).

As shown previously^18–20,39^, mechano-transcription of COX-2 and COX-2 –derived PGE_2_ in gut SMC play a critical role in motility dysfunction in obstruction. The present study suggests that the increased PGE_2_ during bowel distention may further enhance the expression of mPGES-1, which contributes to the sustained increase of PGE_2_ and long-term smooth muscle dysfunction even after distention is corrected. These results indicate that mechano-transcription in the originally distended segment during BO may exert secondary effects on plasticity of colonic SMC, i.e. up-regulation of mPGES-1 by PGE_2_ in an autocrine or paracrine mode. The secondary effects of mechano-transcription contribute to the long-term motility dysfunction. These findings may have important implications for surgical treatment of BO, especially chronic BO such as in HD. The current surgical approach for obstruction is to release blockage or remove constriction (such as in pull-through procedures for HD)^6,9,40^, but to leave alone the distended oral segment in the gut. However, our studies show that the distended oral segment may not be considered “normal”; rather it is the site of mechano-transcription and therefore the root cause of long-term GI dysfunction. The once distended bowel segment during the course of obstruction, may well be the reason for long-term bowel dysfunction even if blockage or constriction has been resolved.

Taken together, we found that gut motility function is suppressed during BO and after BO is surgically resolved. Mechano-transcription of COX-2 and COX-2–derived PGE_2_ in the SMC of the distended bowel segment may not only play a critical role in motility dysfunction during obstruction, but exert secondary effects on SMC to contribute to gut dysfunction even after obstruction is resolved. Our study suggests that COX-2–derived PGE_2_ during BO may further up-regulate the expression of mPGES-1 in an autocrine mode. The increased mPGES-1 contributes to continued production of PGE_2_ and long-term suppression of motility dysfunction in the post-BO state. Thus, the once distended bowel segment during obstruction may not be considered “normal”, as it is where mechano-transcription occurs. Pharmacological studies imply that inhibition of both COX-2 and mPGES-1 during obstruction may be effective as a therapeutic strategy to treat motility dysfunction during BO, and as a prophylactic strategy to prevent long-term motility dysfunction occurring after obstruction is resolved. However, after obstruction is resolved, inhibition of mPGES-1, but not COX-2, may be a more effective therapeutic approach for motility dysfunction.
